# The need to reform our assessment of evidence from clinical trials: A commentary

**DOI:** 10.1186/1747-5341-3-23

**Published:** 2008-09-30

**Authors:** Sean M Bagshaw, Rinaldo Bellomo

**Affiliations:** 1 Division of Critical Care Medicine, University of Alberta Hospital, University of Alberta, Edmonton, Alberta, Canada; 2 Department of Intensive Care, Austin Hospital, Melbourne, Victoria, Australia; 3 Australian and New Zealand Intensive Care Research Centre, Monash University School of Epidemiology and Preventive Medicine, Melbourne, Victoria, Australia

## Abstract

The ideology of evidence-base medicine (EBM) has dramatically altered the way we think, conceptualize, philosophize and practice medicine. One of its major pillars is the appraisal and classification of evidence. Although important and beneficial, this process currently lacks detail and is in need of reform. In particular, it largely focuses on three key dimensions (design, [type I] alpha error and beta [type II] error) to grade the quality of evidence and often omits other crucial aspects of evidence such as biological plausibility, reproducibility, generalizability, temporality, consistency and coherence. It also over-values the randomized trial and meta-analytical techniques, discounts the biasing effect of single centre execution and gives insufficient weight to large and detailed observational studies. Unless these aspects are progressively included into systems for grading, evaluating and classifying evidence and duly empirically assessed (according to the EBM paradigm), the EBM process and movement will remain open to criticism of being more evidence-biased than evidence-based.

"All scientific work is incomplete – whether it be observational or experimental. All scientific work is liable to be upset or modified by advancing knowledge. That does not confer upon us a freedom to ignore the knowledge we already have, or to postpone the action that it appears to demand at a given time".

Sir Bradford Austin Hill [1]

## Introduction

The widespread acceptance of the principles of Evidence-Based Medicine (EBM) have generated a significant paradigm shift in clinical practice, medical education and in how studies are designed, reported, appraised and classified [2,3]. The general principles of EBM are now considered as the golden standard for appraising the quality and strength of evidence created through clinical research [2,3]. These principles also allow for evidence to be classified into different "levels" according to specific characteristics. From these categorical levels of evidence, recommendations are generally issued, each with its own "grade" [4]. (Table 1) These summary recommendations on evidence are then typically used to influence clinical practice through consensus conferences, clinical practice guidelines and systematic reviews or editorials on specific aspects of patient care [5,6].

**Table 1:** Summary of a simplified evidence hierarchy, A) Levels of evidence across clinical research studies, and B) Grading of recommendations based on levels of evidence (Adapted from [2,3])

A)	
Levels of Evidence	

Level I	Well conducted, suitably powered RCT
Level II	Well conducted, but small and under powered RCT
Level III	Non-randomized observational studies
Level IV	Non-randomized study with historical controls
Level V	Case series without controls

B)	

Grades of Recommendations	

Grade A	Level I
Grade B	Level II
Grade C	Level III or lower

In this commentary, we will argue that the present system(s) for classifying the quality of evidence and subsequent formulation of graded recommendations would benefit from reform. A reformed method for classifying evidence should integrate additional dimensions of evidence not traditionally considered, as well as incorporate a method of assigning weight to each dimension when determining the overall quality of the evidence. In this context, we will further comment on the newly proposed hierarchal system, the Grades of Recommendation Assessment, Development and Evaluation (GRADE) system, for gauging the quality of evidence and strength of recommendations from research evidence [7]. The objective of our reflections is to generate further dialogue and discussion about how we currently evaluate evidence from research and how we might improve such evaluation.

### Prediction, truth, and evidence

Ideally, physicians would be able to predict the biological future and clinical outcome of their patients with unbiased certainty and naturally use this knowledge in their care. As an example, physicians would know that the early administration of tissue plasminogen activator (tPA) to a patient with acute massive pulmonary embolism would lead to survival where other interventions would not [8]. Moreover, the physician would also know with certainty that the patient would not suffer any harm as a consequence of having received tPA.

Regrettably, we cannot predict the biological and clinical future with such certainty. Rather, the physician can only be partly reassured by knowing "the operative truth" for questions about this intervention: What would result if all such patients with massive pulmonary embolism were randomly allocated to receive either tPA or an alternative treatment? Would one intervention significantly increase survival over the other? By what magnitude would survival increase? How would such an increase in survival weigh against the potential harms? The physician could then apply knowledge of the "operative truth" about such interventions to guide the course of patient care.

Yet again, such truth in absolute terms is unknown. Rather, physicians are dependent upon estimation and/or measures of probability of the truth for predicting the biological and clinical future of interventions. Naturally, we obtain and apply estimates of the effects of an intervention through the generation of "evidence".

Evidence, can be derived from a multitude of sources: from personal experience, teaching by mentors, local practice patterns, anecdotes, case series, retrospective accounts, prospective observations, non-interventional controlled observations, before-and-after studies, single centre randomized evaluations, randomized evaluation in multiple centres in one or more countries to blinded randomized multi-centre multi-national studies. The evidence generated in each of these forms has both merits and shortcomings. Nonetheless, the focus of this discussion is an examination of how the medical community currently formally appraises, classifies and grades the various forms of evidence.

The process of understanding how new, evolving or "best evidence" is translated into knowledge integrated into patient care remains a great challenge [9,10]. All physicians would generally agree that the provision of high quality care in medicine would, at a minimum, mandate that clinical practice be consistent with the current "best evidence" [11]. Naturally, as a consequence of this notion, numerous evidence hierarchies for classifying and generating recommendations have arisen to aid the busy physician in decisions about management of patients [12]. While they may all have a common theme, to promote the use of "best evidence" in clinical practice, their redundancy may add confusion and threaten to dilute the overall value of EBM [13].

### The evidence hierarchy

The "evidence hierarchy" should emphasize that evidence exists on a continuum of quality. Simply, the evidence generated from some study designs is logically more prone to bias than other designs and, as a consequence, has traditionally provided a weaker justification for influencing clinical practice [13]. Unfortunately, as the levels of evidence have traditionally been expressed as step-wise increases in strength (levels), they have failed to emphasize such continuity.

The apex of the pyramid of evidence has generally been considered the well-conducted and suitably-powered multi-centre multi-national blinded placebo-controlled randomized trial. Such a trial would be characterized by demonstration that intervention X administered to patients with condition A leads to a significant improvement in a clinically-relevant and patient-centred outcome (i.e. survival), when compared to placebo, assuming a genuine and plausible treatment effect of intervention X.

By all current evidence hierarchies, this would be considered as level I evidence that intervention X works for condition A. (Table 1) These findings would generally elicit a strong recommendation (i.e. Grade A) to conclude that intervention X would benefit patients with condition A, assuming no contraindications and that the patients fulfilled all the necessary inclusion/exclusion criteria used to enrol patients in the trial. *Yet, there may be circumstances where a strong recommendation may not be appropriate for such a trial*. This may occur when an intervention does not lead to or is not correlated with improvements in a clinically-relevant patient-centred outcome, when a trial employs, as a primary outcome, a surrogate measure (i.e. physiologic or biochemical endpoint) or when the apparent harm related to an intervention outweighs the benefit. Under these conditions, a lower grade of evidence may be assigned (i.e. Grade B).

In the absence of suitably-powered multi-centre multi-national blinded placebo-controlled randomized trials, many would also regard a high-quality systematic review as level I evidence. Yet, systematic reviews require vigilant interpretation and should not necessarily be considered as high level evidence due to issues related to poor quality, incomplete reporting and the inclusion of evidence from trials of poor quality [14,15]. We contend that systematic reviews/meta-analyses represent an important hypothesis generating activity. However, meta-analysis are not primary evidence, they are statistically assisted interpretations of primary evidence. They have been shown to contradict by confirmatory trials, especially when such meta-analyses are based upon small, low quality studies [16]. We argue that meta-analyses, while perhaps having an important role for the synthesis of previous or current evidence, emphasizing deficiencies and creating a research agenda [17], they should not be used for devising recommendations. As such, should likely be de-emphasized and/or even removed from any classification of evidence in a reformed classification system.

This archetypal hierarchal system would appear reasonable and not in need of reform. Yet, we also contend that traditional hierarchal systems have broadly focused on only three dimensions for defining, classifying and ranking evidence: study design; probability of an alpha or type-I error; and probability of beta or type-II error. We consider these fundamental aspects of trial design for evidence hierarchies below and further discuss a recent initiative (the GRADE system) to improve and standardize how evidence generated from clinical research is classified and graded. Before embarking upon a detailed discussion of the tools used to assess the quality of evidence, we wish to emphasize that no EBM tool can be possibly expected to answer all questions related to evidence. We further notice that a good randomized controlled trial is neither necessary nor sufficient to change practice. However, as we argue below, both are perfectible in specific directions.

### The Grades of Recommendation Assessment, Development and Evaluation (GRADE) System

An updated scheme for grading the quality of evidence and strength of recommendations has been developed by the GRADE Working Group [7,12,18,19]. The primary aim of this informal collaboration was to generate broad consensus for a concise, simplified and explicit classification system that addressed many of the shortcomings of prior hierarchal systems. Moreover, the GRADE Working Group proposed to improve the standardization and transparency of grading evidence and formulating recommendations when translating research evidence into clinical practice.

The GRADE system defines the "quality of evidence" as the amount of confidence that a physician may have that an estimate of effect from research evidence is in fact correct for both beneficial and harmful outcomes [7]. A global judgment on quality requires interrogation of the validity of individual studies through assessment of four key aspects: basic study design (i.e. randomized trial, observational study), quality (i.e. allocation concealment, blinding, attrition rate), consistency (i.e. similarity in results across studies) and directness (i.e. generalizability of evidence). Based on each of these elements and a few other modifying factors, evidence can then be graded as high, moderate, low or very low [7]. (Table 2)

**Table 2:** Overview of the GRADE system for grading the quality of evidence (Adapted from Reference[7]): A) Criteria for assigning grade of evidence; B) Definitions in grading the quality of evidence.

A)	
**Criteria for assigning level of evidence**	

**Type of Evidence**	
Randomized trial	High
Observational study	Low
Any other type of research evidence	Very low

**Increase level if:**	
Strong association	(+1)
Very strong association	(+2)
Evidence of a dose response gradient	(+1)
Plausible confounders reduced the observed effect	(+1)

**Decrease level if:**	
Serious or very serious limitations to study quality	(-1) or (-2)
Important inconsistency	(-1)
Some or major uncertainty about directness	(-1) or (-2)
Imprecise or sparse data*	(-1)
High probability of reporting bias	(-1)

B)	

**Definitions for levels of evidence**	

**High**	Further research is not likely to change our confidence in the effect estimate
**Moderate**	Further research is likely to have an important impact on our confidence in the estimate of effect and may change the estimate
**Low**	Further research is very likely to have an important impact on our confidence in the estimate of effect and is likely to change the estimate
**Very Low**	Any estimate of effect is uncertain

*Few outcome events or observations or wide confident limits around an effect estimate

The "strength of a recommendation" is then defined as the extent in which a clinician can be confident that adherence to the recommendation will result in greater benefit than harm for a patient [7]. Furthermore, there are additional important factors incorporated into the GRADE system that affect the grading of the strength of a recommendation such as target patient population, baseline risk, individual patients' values and costs.

The GRADE system represents a considerable improvement from the traditional hierarchies of grading the quality of evidence and strength of recommendations and has now been endorsed widely by a spectrum of organizations and societies (For details visit: ). Yet, we believe there remain elements of evidence from research that have not been explicitly addressed in the GRADE system and require further consideration and debate as discussed below.

### Traditional measures of the quality

#### Study Design

The design of a clinical trial is a central determinant of its performance and outcome. The principles of EBM would typically focus on several simple key components of study design, such as measures aimed at reducing the probability of bias (i.e. randomization, allocation concealment, blinding). This philosophical stance assumes that the randomized controlled trial represents the "gold standard" as the most scientific and rigorous study design available [20]. Accordingly, for a trial to be classified as level I evidence, it essentially requires incorporation of all of these elements into the design. This approach, while meritorious, often fails to consider additional aspects of study design that warrant attention [21].

First, as an example, in the ARDS Network trial evaluating the impact of low tidal volume ventilation in critically ill patients with acute respiratory distress syndrome (ARDS) on mortality, it now appears that, in the study centers, not all patients allocated to the control group were given a current or near-current accepted therapy or standard of practice. Second, this pivotal trial cannot be easily classified according to the GRADE tool. It is unclear how one can classify trials that assess the implementation of protocols or changes in process of care, which, cannot be blinded [22]. Despite being an unblinded protocol-driven trial, such trials provide the best possible evidence in the field. Assessment of such processes is complex. Clinical trial designs incorporating fixed treatment protocols risk the creation of "practice misalignment". This term refers to the disruption of a fundamental concept in clinical medicine: the relationship between illness severity and the allocated intervention in the control group [23]. The unintended consequence of such trials, as also demonstrated in the ARDS Network Trial and the Transfusion Requirement in Critical Care (TRICC) Trial, may be the formation of non-comparable subgroups across both allocated therapies that potentially lead to harm and generate bias [23-25]. No discussions of these complex interactions between trial design, current practice and adjustment of treatment for illness intensity currently exist or are part of EBM assessment tools.

Second, how can we classify, categorize and compare trials of surgical interventions or devices (i.e. extracorporeal membrane oxygenation (ECMO), high-frequency oscillatory ventilation (HFOV), continuous renal replacement therapy (CRRT)) where true blinding is impossible [22]? Finally, do the study investigators from all centres have genuine clinical equipoise on whether a treatment effect exists across the intervention and control groups? If not, bias could certainly be introduced.

We contend these questions suggest a need for further refinement of how we classify the quality of evidence according to study design. At minimum, this should include principles on how to classify device and "bundle of care" trials and how to incorporate a provision that demonstrates that, as a minimum, the control arm received "standard therapy" (which of itself would require pre-trial evaluation of current practice in the trial centres).

#### Type I Error (Alpha)

A Type I or alpha error describes the probability that a trial would, by chance, find a positive result for an intervention (i.e. effective) when, in fact, it is not (false-positive) and represent a chance or statistical error. In general, the alpha value for any given trial has traditionally and somewhat arbitrary been set at < 0.05. While recent trends have brought greater recognition for hypothesis testing by use of confidence intervals, the use of an alpha value remains common for statistical purposes and sample size estimation in trial design.

The possibility of a type I error is generally inversely related to study sample size. Thus, a study with a small sample size or relatively small imbalances across allocated groups or in the context of numerous interim analyses might be sufficient, alone or together, to lead to detectable differences in outcome not attributable to the intervention. Likewise, a trial with few observed outcome events, often resulting in wide confidence limits around an effect estimate, will potentially be prone to such an error.

The potential bias due to type I errors can be recognized by evaluation of key aspects of the study design and findings. These include whether the trial employed a patient-centred or surrogate measure as the primary outcome, evaluation of the strength of association between the intervention and primary outcome (i.e. relative risk or odds ratio), assessment of the precision around the effect estimate (i.e. confidence limits), and a determination of the baseline or control group observed event rate. Level I evidence mandates that trials have a low probability of committing a type I error. While desirable, how do we clinically or statistically measure a given trial's probability of type I error? Should we adjust the statistical significance of an intervention found in a clinical trial to the probability of a type I error? These questions suggest a need for both discussion and consensus on the concept of alpha error and its practical application. This discussion has not formally taken place in the literature.

#### Type II error (Beta)

A type II or beta error describes a statistical error where a trial would find that an intervention is negative (i.e. not effective) when, in fact, it is not (false-negative). An increase in sample size and the number of observed outcome events reduce the probability a type II error, on the assumption that a genuine difference in effect exists across the allocated groups. Thus, to minimize the chance of a type II error, clinical trials must be suitably "powered". In general, the probability of type II error is conventionally and arbitrarily set at 0.10–0.20 (i.e. power 0.80–0.90). The calculation of power is used in study design to determine and justify the overall sample size. Inadequately powered trials risk missing small but potentially important clinically differences in outcome attributable to the intervention under assessment [26,27]. Naturally, the ideal trial is one in which the power is high. Yet, while maximizing the power of a trial may appear logical, such an increase has both ethical and cost implications [28]. For a given increase in power (i.e. from 0.20 to 0.10), trial recruitment would correspondingly need to increase, potentially exposing a larger cohort of patients to a placebo intervention and certainly leading to an increase in trial costs.

Given these implications, should attaining suitable power for a trial simply be a matter of statistical consideration? Can we standardize what suitable power represents for a given intervention? Should we subject suitable power in trial design to additional public health considerations such as: the size of the population likely to benefit from the intervention if proven effective; the clinical relevance of the outcome being assessed; and the potential downstream cost of integrating the intervention in clinical practice? We also contend that these issues warrant consideration in the context of trials of equivalency or non-superiority and more specifically for trials that are pre-maturely terminated at interim analyses [29-31]. Finally, we believe that future trial design should address whether estimates of risk reduction used to justify sample size calculations for an intervention are biologically plausible, are supported by previous preliminary evidence and are truly feasible while considering the aforementioned issues.

### Additional insights and considerations

In 1965, Sir Austin Bradford Hill described nine issues he considered important, in a Presidential Address to the Section of Occupation Medicine of the Royal Society of Medicine, for potentially inferring causation from statistical associations observed in epidemiology data [1]. (Table 3) These considerations were not simply intended as "criteria" as has been widely interpreted, but instead as a pragmatic and philosophical method to assess the potential for affecting our confidence in concluding causality. Hill quoted: "None of my nine viewpoints can bring indisputable evidence for or against the cause-and-effect hypothesis and none can be required as a *sine qua non*". Above, we considered and discussed the limitations of several traditional measures to evaluate the quality of evidence, in particular with a focus on what EBM considers the "old standard" – randomized trials. Below, we contend that there are additional dimensions to evidence that merit attention, both for randomized trials and epidemiologic studies, when appraising, classifying and grading the quality of evidence. Moreover, many of these dimensions take into account the issues identified by Hill when deciding whether an observed association was a causal relationship.

**Table 3:** Aspects of association to consider prior to the provisional inference of causation as proposed by Sir Austin Bradford Hill (Adapted from [1])

**Criteria**	**Description**
Strength	Correlation or relative measures of effect (i.e. risk ratio)
Consistency	Across variable studies in design, populations, settings, circumstances, and time
Specificity	Intervention causes the effect
Temporality	Intervention precedes effect
Biologic Gradient	Dose-response curve between intervention and effect
Plausibility	Based on the current biologic knowledge of mechanisms of disease
Coherence	In the context of knowledge of natural history and related treatments
Experiment	Prospective clinical investigations of hypotheses

Evidence from randomized trials does not and cannot stand on its own, independent of previous information or studies. As discussed previously, prior knowledge can be accrued from a variety of sources ranging from personal experience to *in vitro *studies, experimental data, epidemiologic investigations and additional randomized trials. In fact, there may be circumstances for which randomized trials are unnecessary (i.e. due to obvious and large treatment effect), or more importantly, unethical [20,21]. For instance, there is an extensive list of historical examples of widely accepted and uncontested interventions that are based solely on case-series and non-randomized cohort studies. (Table 4) By all reasonable consideration, no human research ethics board would support a randomized trial that compared insulin to placebo for patients with new onset type I diabetes mellitus or a randomized comparison of neostigmine versus placebo for initial therapy of proven myesthenia gravis [32,33]. Accordingly, the systematic methods for how we evaluate and classify evidence need to consider these circumstances.

**Table 4:** Selected historical examples of interventions widely endorsed and seldom contested that are not based on any evidence from randomized trials. (Adapted from [63])

**Intervention**
Blood transfusion for severe hemorrhagic shock
Defibrillation for ventricular fibrillation or pulseless ventricular tachycardia
Neostigmine for myasthenia gravis
Suturing for repair of large wounds
Closed reduction/splinting for displaced long-bone fractures
Insulin for diabetes mellitus
Directed pressure/suturing to stop bleeding
Activated charcoal for strychnine poisoning

More recently, there have been high profile examples of large epidemiologic studies (phase IV studies) of interventions showing previously unknown potential harm [34-38]. This represents one important aspect of Hill's philosophy that has often been neglected, specifically, the postponing of action or the dismissing altogether of new data due to its limitations of potential harm associated with interventions. Similarly, we need to consider a means for incorporating the evolution of evidence and these additional aspects, such as harm, outside of the usual realm of randomized trials.

#### Biological plausibility, temporality, and coherence

These issues were central to Hill's viewpoints and, while seemingly obvious, are in fact not always evident. For example, most, perhaps all, reasonable clinicians would reject the findings of a randomized trial of retroactive intercessory prayer compared with usual care showing a statistically significant decrease in the duration of hospital stay in patients with septicemia [39]. Such a study completely lacks biological plausibility, along with rejecting the tenets of temporality and coherence [40]. On the other hand, perhaps fewer physicians would have rejected the findings of the first interim analysis of the AML UK MRC study of 5 courses of chemotherapy compared to 4, where the investigators showed a 53% decrease in the odds of death (odds ratio 0.47; 95% CI, 0.29–0.77, p = 0.003) [31]. Yet the data safety and monitoring committee decided to continue the trial because these initial findings were considered *too large to be clinically possible*, and *lacked biological plausibility and coherence*. Accordingly, the committee recommended the trial be continued and the final results (no difference between the two therapies) vindicated this apparent chance finding at interim analysis [31]. These examples both afford an opportunity to highlight how the results of randomized trials can be influenced by chance statistical findings, however improbable, and further deviate from the current and recognized knowledge of the day. To date, there has been no formal incorporation of "biological plausibility" into the grading of the quality of evidence or strength of recommendations. *We believe this dimension, along with issues of temporality and coherence, should be more formally acknowledged in a reformed classification system*. We similarly believe that careful attention must be paid to *robust *findings which contradict current beliefs and concepts of what is biologically plausible.

#### Consistency and applicability

Consistency in evidence refers to finding reproducibility in the effect of an intervention in numerous studies and across diverse populations, settings, and time. For example, the PROWESS trial tested the efficacy of rhAPC in severe sepsis, however, it was limited in scope by the study inclusion criteria (i.e. adults, weight < 135 kg, age > 18 years, number of organ failures etc.) [41]. Yet, the evidence for a similar beneficial effect of rhAPC in additional studies enrolling different populations, in different settings and under different circumstances has been remarkably less certain [42-44]. Accordingly, rhAPC has lacked consistency in the treatment of sepsis. In addition, we also need to consider the extraordinary cost of rhAPC. The expense makes its applicability outside of wealthy industrialized countries unfeasible and more likely near impossible [45,46]. While the cost of an intervention clearly has no bearing on the quality of such evidence, it has major relevance to its applicability outside of rich countries, where such treatments are nonetheless heavily promoted by drug companies. This "population relevance" limitation could be similarly applied to numerous innovations in medical interventions and can be usefully incorporated in a grading tool.

Likewise, "complex interventions" which involve devices, therapies, protocols or processes (i.e. high-frequency oscillatory ventilation, continuous renal replacement therapy [CRRT], intensive insulin therapy or medical emergency teams, early-goal direct therapy for severe sepsis) pose major challenges for researchers [22,32]. The findings of such trials, if negative, often require consideration of whether the "intervention" was ineffective, whether the "intervention" was inadequately applied or applied in an inappropriate method, or whether the trial used an unsuitable design, selected an inappropriate patient population or used the wrong outcome measures for determining effect [32]. Conversely, if the "complex intervention" leads to a beneficial effect, further challenges arise for how to apply such data in a broader context. In addition, examples of "complex interventions" as applied in a given trial often imply or assume equity across an entire infrastructure of medical, surgical and nursing availability, knowledge, expertise and logistics [47-51]. Yet, such equity is far less common than appreciated. Moreover, such interventions are often not universally available. Thus, the translation of a "complex intervention" in isolation to a setting outside of its initial development may have both negative health and economic consequences. Similarly, one can present a moral and ethical argument regarding the vast resources utilized for the development and evaluation of interventions that are likely to benefit very few and reach even fewer patients.

We contend that due thought needs to be given to how the results of a trial can be translated into interventions that reliably work, are reproducible, are broadly applicable and can be applied elsewhere. The GRADE system does incorporate a subjective assessment of consistency as criteria for grading the quality of evidence and, in the setting of unexplained heterogeneity across trials, suggests a decrease in grade [7]. *We consider that a formal grading of applicability is needed in future classifications of evidence*.

#### Generalizability

The generalizability of findings from a clinical trial represents a fundamental dimension of evidence, that of external validity. Narrow controls designed to optimize the internal validity of a trial (i.e. inclusion/exclusion criteria, intervention protocol) can compete with and compromise overall generalizability [21,52]. Whether an individual trial is widely generalizable can also be the result of additional factors. For example, the power of a local investigator-protagonist needs to be taken into account. Such investigators, when involved in single centre studies, especially unblinded ones, have the power to profoundly influence outcome and behaviour through their commitment to the cause, expertise, dedication and enthusiasm. Examples of such studies include use of early-goal directed therapy, higher volume CRRT, or tight glycemic control [47,49,51]. All these studies were single centre evaluation of complex interventions, but importantly, all had a local protagonist. Alternatively, the findings of a multi-centre trial of an intervention may not be generalizable if only large tertiary/academic centres were involved, where there may be a natural predilection to selection bias.

How generalizable are the findings of a single centre study, however well designed? Should single centre trials ever lead to level I evidence or grade A recommendations? Accordingly, how should we classify the evidence from a single centre trial showing benefit? For example, would early goal-directed resuscitation really improve the outcome of all patients with septic shock presenting to Emergency Departments worldwide or do the findings of this trial simply reflect improvements in patient care in a single institution where there existed a very high pre-intervention mortality [51]? These are more than idle questions because numerous single centre studies have profoundly influenced and are continuing to influence the practice of critical care medicine worldwide and have been incorporated in international guidelines [53]. Yet, two recent assessments of interventions that in single centre studies looked extraordinarily promising (i.e. steroids for the fibro-proliferative phase of ARDS and introduction of a Medical Emergency Response Team), failed to show a benefit when evaluated in a multi-centre setting [48,54].

In the end, there needs to be a greater understanding and consensus around the limitations of data from single centre studies. We need to consider the meaning of multi-centre and how it relates to grading the quality of evidence. Additionally, we need to consider and discuss the implications of multi-centre studies sponsored by industry that evaluate new pharmaceutical interventions. We also need to relate the control population studied in any single or multi-centre trial to other large populations with respect to the same condition, so that we can consider the "generalizability level" of a given study.

Importantly, we also need to give a greater consideration to the weight of evidence from observational studies in the context of the known limitations of randomized trials [20,55,56]. While randomized trials are certainly the most ideal study design in some circumstances, in other cases observational studies may in fact be more feasible and accurate. Well-conducted observational studies have a pivotal role in the generation of high-quality research evidence and not only serve as an adjuvant to the data generated from randomized trials. Such observational studies may enable better "real world" estimates of the impact of an intervention (including potential harm) compared with that of a randomized trial of the same intervention which enrolled patients within tight inclusion/exclusion criteria [57]. Why do we, by default, rank the randomized trial higher on current classification scales? How do we empirically know it to be more robust evidence? Where are the studies testing how many very large observational studies have been refuted or confirmed by subsequent large randomized controlled trial compared with single centre randomized controlled trials? The recent trial-based confirmation of the risks associated with aprotinin during cardiopulmonary bypass and the related FDA alert (For details visit: ) arose from observational data and were missed in single centre studies [34,35,38,57,58]. Even more powerfully, such single centre studies had led to widespread prescription of beta-blockers in high-risk patients receiving non cardiac surgery [59]. The recent POISE trial of > 8000 patients demonstrated that such prescription actually increases mortality [60].

While there are obvious differences in study design, well-performed observational studies may provide a powerful mechanism to improve the generalizability of evidence and may well provide more robust evidence than single centre randomized controlled trials [20,21,55,56,61,62]. Randomized trials, especially if evaluating complex interventions or with strict inclusion/exclusion criteria, often only provide data in a clinical context that does not exist outside the trial itself and have limited power to detect harm. Importantly, observational studies have the distinct advantage of examining the long-term effects or prognosis of an intervention and, as discussed above, evaluating for adverse or rare outcome events [34-37]. We contend work needs to be done to evaluate how prior observational studies perform in comparison with small or single centre randomized trials in their ability to detect an effect and which was subsequently confirmed in at least one large multi-centre randomized trial. It may well be that such studies might show that observational studies with appropriate detailed variable collection and statistical correction are statistically more likely to detect beneficial effects or harm than small or single centre studies. If this were the case, objective evidence would exist to reform a classification system, which was not yet considered this issue.

### The need for further reform and consensus

An argument can be made that proposed classification schemes, especially the new GRADE system, are best left alone. They are reasonably simple, explicit, have been validated and now are increasingly endorsed. Furthermore, the additional dimensions of evidence we have discussed (i.e. study design, biological plausibility, coherence, consistency, and generalizability) are often difficult to simply measure and their impact on how the findings of an individual trial approximate the "truth" is hard to quantify. (Table 5) On the other hand, we believe these issues are valid and deserve broader discussion, consideration and debate.

**Table 5:** Summary of components to consider when evaluating the quality of evidence from research.

**Components**
**Study Design:**
Randomized
Allocation concealment
Blinding (if possible)*
Clinically important and objective primary outcome
Beta-error**
Multi-centre

**Study Conduct:**
Intention-to-treat analysis
Follow-up or attrition rate
Completion to planned numbers

**Study Findings:**
Biological plausibility
Strength of estimate of effect
Precision of estimate of effect
Observed event rate

**Study Applicability:**
Complex intervention
Consistency across similar studies
Generalizability
Cost of intervention

*Blinding may not be possible in device or protocol/process trials

**Adequately powered, appropriate estimate of control event rate and relative or absolute reduction in patient-centred and clinically important primary outcome.

A classification system which is simple is indeed desirable but becomes a problem when, for the sake of simplicity, it fails to take into account important aspects of the growing complexity of the evidence available. Accordingly, summary classifications of the quality of evidence and strength of recommendations, such as the GRADE system, will continue to have an important and expanding role in medicine. We believe that as the GRADE system becomes more widely endorsed, additional refinements to the system will result in appropriate recognition of higher quality evidence and contribute to greater confidence in recommendations for clinical practice. We also believe that this field is very much "work in progress" and needs to evolve more explicit recognition and classification of the dimensions of trial design discussed in this manuscript.

## Conclusion

In this commentary, we have argued in favour of the concept that assessing of the quality and strength of evidence from clinical studies requires reform. Such reform should, in particular, reflect those dimensions of evidence, which are currently not routinely or explicitly addressed. The GRADE Working Group has made considerable contributions to improving how the quality of research evidence and recommendations are graded. We believe that additional reform is needed to explicitly address and quantify dimensions of evidence such as biological plausibility, reproducibility, temporality, consistency, ability to detect harm and generalizability. We also believe that observational studies need to be graded and that, under some circumstances, such studies provide either evidence that cannot be detected in single centre studies or even better evidence than produced from small, randomised trials. We believe such reform should occur through consensus. We also believe that such reform will have lasting beneficial effects on clinical practice and on the future design, reporting and assessment of clinical studies.

## Authors' contributions

Both authors (SMB and RB) wrote and revised the manuscript. Both authors read and approved the final manuscript.

## About the authors

**Dr. Sean Bagshaw **is an intensivist at the University of Alberta Hospital and an Assistant Professor in the Division of Critical Care Medicine, University of Alberta, Edmonton, Canada. His research interests include acute kidney injury and extracorporeal therapies in critically ill patients, medical emergency/rapid response teams, end-of-life care and clinical trial/meta-analysis methodology.

**Prof. Rinaldo Bellomo **is Professor of Medicine with Melbourne, Monash and Sydney University and Director of Intensive Care Research at the Austin Hospital, Melbourne. He has been on the management committee of all the multicentre randomized controlled trials conducted by the Australian and New Zealand Intensive Care Society Clinical Trials Group (patients randomized = 14,921).

## References

[B1] Hill AB (1965). The Environment and Disease: Association or Causation?. Proc R Soc Med.

[B2] Cook DJ, Guyatt GH, Laupacis A, Sackett DL (1992). Rules of evidence and clinical recommendations on the use of antithrombotic agents. Chest.

[B3] Cook DJ, Guyatt GH, Laupacis A, Sackett DL, Goldberg RJ (1995). Clinical recommendations using levels of evidence for antithrombotic agents. Chest.

[B4] Guyatt GH, Cook DJ, Sackett DL, Eckman M, Pauker S (1998). Grades of recommendation for antithrombotic agents. Chest.

[B5] Bellomo R, Ronco C, Kellum JA, Mehta RL, Palevsky P (2004). Acute renal failure – definition, outcome measures, animal models, fluid therapy and information technology needs: the Second International Consensus Conference of the Acute Dialysis Quality Initiative (ADQI) Group. Crit Care.

[B6] Dellinger RP, Carlet JM, Masur H, Gerlach H, Calandra T, Cohen J, Gea-Banacloche J, Keh D, Marshall JC, Parker MM (2004). Surviving Sepsis Campaign guidelines for management of severe sepsis and septic shock. Crit Care Med.

[B7] Atkins D, Best D, Briss PA, Eccles M, Falck-Ytter Y, Flottorp S, Guyatt GH, Harbour RT, Haugh MC, Henry D (2004). Grading quality of evidence and strength of recommendations. Bmj.

[B8] Konstantinides S, Geibel A, Heusel G, Heinrich F, Kasper W (2002). Heparin plus alteplase compared with heparin alone in patients with submassive pulmonary embolism. N Engl J Med.

[B9] Upshur RE (2001). The ethics of alpha: reflections on statistics, evidence and values in medicine. Theor Med Bioeth.

[B10] Ferreira F, Vincent JL, Brun-Buisson C, Sprung C, Sibbald W, Cook D (2001). Doctors' perceptions of the effects of interventions tested in prospective, randomised, controlled, clinical trials: results of a survey of ICU physicians. Intensive Care Med.

[B11] Guyatt GH, Meade MO, Jaeschke RZ, Cook DJ, Haynes RB (2000). Practitioners of evidence based care. Not all clinicians need to appraise evidence from scratch but all need some skills. Bmj.

[B12] Atkins D, Eccles M, Flottorp S, Guyatt GH, Henry D, Hill S, Liberati A, O'Connell D, Oxman AD, Phillips B (2004). Systems for grading the quality of evidence and the strength of recommendations I: critical appraisal of existing approaches The GRADE Working Group. BMC Health Serv Res.

[B13] Upshur RE (2003). Are all evidence-based practices alike? Problems in the ranking of evidence. Cmaj.

[B14] Delaney A, Bagshaw SM, Ferland A, Manns B, Laupland KB, Doig CJ (2005). A systematic evaluation of the quality of meta-analyses in the critical care literature. Crit Care.

[B15] Biondi-Zoccai GG, Lotrionte M, Abbate A, Testa L, Remigi E, Burzotta F, Valgimigli M, Romagnoli E, Crea F, Agostoni P (2006). Compliance with QUOROM and quality of reporting of overlapping meta-analyses on the role of acetylcysteine in the prevention of contrast associated nephropathy: case study. Bmj.

[B16] LeLorier J, Gregoire G, Benhaddad A, Lapierre J, Derderian F (1997). Discrepancies between meta-analyses and subsequent large randomized, controlled trials. N Engl J Med.

[B17] Young C, Horton R (2005). Putting clinical trials into context. Lancet.

[B18] Atkins D, Briss PA, Eccles M, Flottorp S, Guyatt GH, Harbour RT, Hill S, Jaeschke R, Liberati A, Magrini N (2005). Systems for grading the quality of evidence and the strength of recommendations II: pilot study of a new system. BMC Health Serv Res.

[B19] Schunemann HJ, Best D, Vist G, Oxman AD (2003). Letters, numbers, symbols and words: how to communicate grades of evidence and recommendations. Cmaj.

[B20] Grossman J, Mackenzie FJ (2005). The randomized controlled trial: gold standard, or merely standard?. Perspect Biol Med.

[B21] Mercer SL, DeVinney BJ, Fine LJ, Green LW, Dougherty D (2007). Study designs for effectiveness and translation research: identifying trade-offs. Am J Prev Med.

[B22] Delaney A, Angus DC, Bellomo R, Cameron P, Cooper DJ, Finfer S, Harrison DA, Huang DT, Myburgh JA, Peake SL (2008). Bench-to-bedside review: The evaluation of complex interventions in critical care. Crit Care.

[B23] Deans KJ, Minneci PC, Suffredini AF, Danner RL, Hoffman WD, Ciu X, Klein HG, Schechter AN, Banks SM, Eichacker PQ, Natanson C (2007). Randomization in clinical trials of titrated therapies: unintended consequences of using fixed treatment protocols. Crit Care Med.

[B24] (2000). Ventilation with lower tidal volumes as compared with traditional tidal volumes for acute lung injury and the acute respiratory distress syndrome. The Acute Respiratory Distress Syndrome Network. N Engl J Med.

[B25] Hebert PC, Wells G, Blajchman MA, Marshall J, Martin C, Pagliarello G, Tweeddale M, Schweitzer I, Yetisir E (1999). A multicenter, randomized, controlled clinical trial of transfusion requirements in critical care. Transfusion Requirements in Critical Care Investigators, Canadian Critical Care Trials Group. N Engl J Med.

[B26] Moher D, Dulberg CS, Wells GA (1994). Statistical power, sample size, and their reporting in randomized controlled trials. Jama.

[B27] Halpern SD, Karlawish JH, Berlin JA (2002). The continuing unethical conduct of underpowered clinical trials. Jama.

[B28] Whitley E, Ball J (2002). Statistics review 4: sample size calculations. Crit Care.

[B29] Montori VM, Devereaux PJ, Adhikari NK, Burns KE, Eggert CH, Briel M, Lacchetti C, Leung TW, Darling E, Bryant DM (2005). Randomized trials stopped early for benefit: a systematic review. Jama.

[B30] Le Henanff A, Giraudeau B, Baron G, Ravaud P (2006). Quality of reporting of noninferiority and equivalence randomized trials. Jama.

[B31] Wheatley K, Clayton D (2003). Be skeptical about unexpected large apparent treatment effects: the case of an MRC AML12 randomization. Control Clin Trials.

[B32] Banting FG, Best CH, Collip JB, Campbell WR, Fletcher AA (1991). Pancreatic extracts in the treatment of diabetes mellitus: preliminary report. 1922. Cmaj.

[B33] Walker MB (1973). Some discoveries on myasthenia gravis: the background. Br Med J.

[B34] Mangano DT, Miao Y, Vuylsteke A, Tudor IC, Juneja R, Filipescu D, Hoeft A, Fontes ML, Hillel Z, Ott E (2007). Mortality associated with aprotinin during 5 years following coronary artery bypass graft surgery. Jama.

[B35] Mangano DT, Tudor IC, Dietzel C (2006). The risk associated with aprotinin in cardiac surgery. N Engl J Med.

[B36] Nissen SE, Wolski K (2007). Effect of rosiglitazone on the risk of myocardial infarction and death from cardiovascular causes. N Engl J Med.

[B37] Bavry AA, Kumbhani DJ, Helton TJ, Borek PP, Mood GR, Bhatt DL (2006). Late thrombosis of drug-eluting stents: a meta-analysis of randomized clinical trials. Am J Med.

[B38] Shaw AD, Stafford-Smith M, White WD, Phillips-Bute B, Swaminathan M, Milano C, Welsby IJ, Aronson S, Mathew JP, Peterson ED, Newman MF (2008). The effect of aprotinin on outcome after coronary-artery bypass grafting. N Engl J Med.

[B39] Leibovici L (2001). Effects of remote, retroactive intercessory prayer on outcomes in patients with bloodstream infection: randomised controlled trial. Bmj.

[B40] Hettiaratchy S, Hemsley C (2002). Effect of retroactive intercessory prayer. Paper proves power of statistics, not prayer. Bmj.

[B41] Bernard GR, Vincent JL, Laterre PF, LaRosa SP, Dhainaut JF, Lopez-Rodriguez A, Steingrub JS, Garber GE, Helterbrand JD, Ely EW, Fisher CJ (2001). Efficacy and safety of recombinant human activated protein C for severe sepsis. N Engl J Med.

[B42] Nadel S, Goldstein B, Williams MD, Dalton H, Peters M, Macias WL, Abd-Allah SA, Levy H, Angle R, Wang D (2007). Drotrecogin alfa (activated) in children with severe sepsis: a multicentre phase III randomised controlled trial. Lancet.

[B43] Abraham E, Laterre PF, Garg R, Levy H, Talwar D, Trzaskoma BL, Francois B, Guy JS, Bruckmann M, Rea-Neto A (2005). Drotrecogin alfa (activated) for adults with severe sepsis and a low risk of death. N Engl J Med.

[B44] Fry DE, Beilman G, Johnson S, Williams MD, Rodman G, Booth FV, Bates BM, McCollam JS, Lowry SF (2004). Safety of drotrecogin alfa (activated) in surgical patients with severe sepsis. Surg Infect (Larchmt).

[B45] Chalfin DB, Teres D, Rapoport J (2003). A price for cost-effectiveness: implications for recombinant human activated protein C (rhAPC). Crit Care Med.

[B46] Manns BJ, Lee H, Doig CJ, Johnson D, Donaldson C (2002). An economic evaluation of activated protein C treatment for severe sepsis. N Engl J Med.

[B47] Berghe G van den, Wouters P, Weekers F, Verwaest C, Bruyninckx F, Schetz M, Vlasselaers D, Ferdinande P, Lauwers P, Bouillon R (2001). Intensive insulin therapy in the critically ill patients. N Engl J Med.

[B48] Hillman K, Chen J, Cretikos M, Bellomo R, Brown D, Doig G, Finfer S, Flabouris A (2005). Introduction of the medical emergency team (MET) system: a cluster-randomised controlled trial. Lancet.

[B49] Ronco C, Bellomo R, Homel P, Brendolan A, Dan M, Piccinni P, La Greca G (2000). Effects of different doses in continuous veno-venous haemofiltration on outcomes of acute renal failure: a prospective randomised trial. Lancet.

[B50] Derdak S, Mehta S, Stewart TE, Smith T, Rogers M, Buchman TG, Carlin B, Lowson S, Granton J (2002). High-frequency oscillatory ventilation for acute respiratory distress syndrome in adults: a randomized, controlled trial. Am J Respir Crit Care Med.

[B51] Rivers E, Nguyen B, Havstad S, Ressler J, Muzzin A, Knoblich B, Peterson E, Tomlanovich M (2001). Early goal-directed therapy in the treatment of severe sepsis and septic shock. N Engl J Med.

[B52] Green LW, Glasgow RE (2006). Evaluating the relevance, generalization, and applicability of research: issues in external validation and translation methodology. Eval Health Prof.

[B53] Dellinger RP, Levy MM, Carlet JM, Bion J, Parker MM, Jaeschke R, Reinhart K, Angus DC, Brun-Buisson C, Beale R (2008). Surviving Sepsis Campaign: international guidelines for management of severe sepsis and septic shock: 2008. Crit Care Med.

[B54] Steinberg KP, Hudson LD, Goodman RB, Hough CL, Lanken PN, Hyzy R, Thompson BT, Ancukiewicz M (2006). Efficacy and safety of corticosteroids for persistent acute respiratory distress syndrome. N Engl J Med.

[B55] Blair E (2004). Gold is not always good enough: the shortcomings of randomization when evaluating interventions in small heterogeneous samples. J Clin Epidemiol.

[B56] Sanson-Fisher RW, Bonevski B, Green LW, D'Este C (2007). Limitations of the randomized controlled trial in evaluating population-based health interventions. Am J Prev Med.

[B57] Hiatt WR (2006). Observational studies of drug safety – aprotinin and the absence of transparency. N Engl J Med.

[B58] US Food and Drug Administration (FDA) (2007). Early Communication about an Ongoing Safety Review Aprotinin Injection (marketed as Trasylol). Center for Drug Evaluation and Research.

[B59] Mangano DT, Layug EL, Wallace A, Tateo I (1996). Effect of atenolol on mortality and cardiovascular morbidity after noncardiac surgery. Multicenter Study of Perioperative Ischemia Research Group. N Engl J Med.

[B60] POISE Investigators (2008). Effects of extended-release metoprolol succinate in patients undergoing non-cardiac surgery (POISE trial): a randomised controlled trial. Lancet.

[B61] Herman J (1998). Shortcomings of the randomized controlled trial: a view from the boondocks. J Eval Clin Pract.

[B62] Simon SD (2001). Is the randomized clinical trial the gold standard of research?. J Androl.

[B63] Glasziou P, Vandenbroucke JP, Chalmers I (2004). Assessing the quality of research. Bmj.

